# Obstructive Sleep Apnea and Gastroesophageal Reflux Disease: A Systematic Review and Meta-Analysis of Observational Studies

**DOI:** 10.3390/jcm15072518

**Published:** 2026-03-26

**Authors:** Marines Vega Sanchez, Francisco Córdova, Verónica Mosquera Cisneros, Maria Rodríguez Tates, Luis Chauca Bajaña, Diego Quiguango Farias, María Flores Araque, Byron Velasquez Ron

**Affiliations:** 1 Carrera de Odontología, Universidad de Las Américas (UDLA) Ecuador, Av. Colón y 6. Diciembre CP, Quito 170516, Ecuador or marinesvega01@gmail.com (M.V.S.); francisco.cordova@udla.edu.ec (F.C.); gaby.rodriguezt1@gmail.com (M.R.T.); maria.flores.araque@udla.edu.ec (M.F.A.); 2 Carrera de Odontología, Grupo de Investigación Epidemiológica y Clínica en Odontología, Universidad Politécnica Salesiana (UPS), Cuenca 010105, Ecuador; vmosquera@ups.edu.ec; 3 Dental Sciences, College Dentistry, University of Guayaquil, Guayaquil 090514, Ecuador; luischauk@hotmail.com; 4 Carrera Ciencias de la Salud, Maestría en Salud Publica, Universidad de Las Américas (UDLA) Ecuador, Quito 170516, Ecuador; diego.quiguango@udla.edu.ec

**Keywords:** sleep apnea, obstructive, gastroesophageal reflux, meta-analysis, systematic review, observational studies, risk factors

## Abstract

**Background**: Obstructive sleep apnea (OSA) is a prevalent sleep-related breathing disorder associated with multiple systemic comorbidities. Gastroesophageal reflux disease (GERD) has been frequently reported among patients with OSA; however, the magnitude of this association remains uncertain. **Objective**: To systematically evaluate and quantify the association between obstructive sleep apnea and gastroesophageal reflux disease in adult populations. **Methods**: A systematic review and meta-analysis were conducted in accordance with PRISMA guidelines and a protocol registered in PROSPERO (CRD420261278563). Electronic searches were performed in PubMed/MEDLINE, Scopus, Web of Science, and Embase. Observational studies assessing the association between OSA and GERD were included. Risk of bias was assessed using the Newcastle–Ottawa Scale. A random-effects meta-analysis was performed to pool odds ratios (ORs) with 95% confidence intervals (CIs). Results: Six observational studies were included in the quantitative synthesis. The pooled analysis demonstrated a statistically significant association between obstructive sleep apnea and gastroesophageal reflux disease, with a combined OR of 1.96. Moderate heterogeneity was observed among studies (I^2^ = 65%). No substantial evidence of publication bias was identified based on funnel plot assessment. **Conclusions**: These findings suggest that obstructive sleep apnea is associated with nearly two-fold increased odds of gastroesophageal reflux disease and support increased clinical awareness rather than formal screening recommendations.

## 1. Introduction

Obstructive sleep apnea (OSA) is a highly prevalent sleep-related breathing disorder characterized by recurrent episodes of upper airway obstruction during sleep, leading to intermittent hypoxia, sleep fragmentation, and excessive daytime sleepiness. OSA has been increasingly recognized as a systemic condition with significant cardiometabolic, neurocognitive, and gastrointestinal consequences, rather than an isolated respiratory disorder [1,2,3]. OSA is estimated to affect approximately 9–38% of the adult population globally, with higher prevalence in males and individuals with obesity. Moderate-to-severe OSA affects approximately 6–17% of middle-aged adults. Among its extracardiac comorbidities, gastroesophageal reflux disease (GERD) has attracted growing clinical and research interest. GERD is a chronic gastrointestinal disorder defined by the reflux of gastric contents into the esophagus, resulting in typical symptoms such as heartburn and regurgitation, as well as extraesophageal manifestations. The prevalence of GERD has risen worldwide, paralleling increases in obesity and metabolic disorders, which are also established risk factors for OSA [4,5]. This epidemiological overlap has prompted investigation into potential bidirectional mechanisms linking OSA and GERD. Several pathophysiological pathways have been proposed to explain the association between OSA and GERD. Negative intrathoracic pressure generated during obstructive apneic events may promote gastric content reflux by increasing the trans-diaphragmatic pressure gradient [6]. Additionally, sleep fragmentation and autonomic dysregulation in OSA may impair esophageal motility and lower esophageal sphincter function [7]. Obesity, particularly central adiposity, further contributes to both conditions by increasing intra-abdominal pressure and altering respiratory mechanics [8]. Observational studies have consistently reported a higher prevalence of GERD symptoms and endoscopically confirmed reflux disease among patients with OSA, as well as an association between OSA severity and GERD risk [9,10,11,12,13]. Conversely, GERD-related nocturnal symptoms may exacerbate sleep disruption, suggesting a potentially reinforcing relationship between the two disorders [14]. Despite these observations, reported effect sizes vary across studies, and differences in study design, population characteristics, diagnostic criteria, and adjustment for confounding factors have limited the ability to draw definitive conclusions. To date, evidence regarding the magnitude and consistency of the association between OSA and GERD remains fragmented [15,16]. A comprehensive synthesis of available observational data is therefore warranted to clarify this relationship and quantify the pooled effect estimates [17]. Accordingly, the present systematic review and meta-analysis aimed to evaluate the association between obstructive sleep apnea and gastroesophageal reflux disease in adult populations, integrating available observational evidence and assessing methodological quality and risk of bias.

## 2. Methods

### 2.1. Protocol Registration and Reporting Guidelines

This systematic review and meta-analysis were conducted in accordance with the Preferred Reporting Items for Systematic Reviews and Meta-Analyses (PRISMA) guidelines. The methodological approach was guided by a protocol registered in the International Prospective Register of Systematic Reviews (PROSPERO; registration number: CRD420261278563), which defined the research question, eligibility criteria, and analytical strategy a priori. The search strategy included combinations of the following terms: (“obstructive sleep apnea” OR “OSA” OR “sleep-disordered breathing”) AND (“gastroesophageal reflux” OR “GERD” OR “non-erosive reflux disease” OR “reflux symptoms”).

### 2.2. PRISMA Flow Diagram

The study selection process was conducted in accordance with the PRISMA 2020 guidelines. Records were identified through electronic database searches in PubMed/MEDLINE, Scopus, Web of Science, and Embase. After removal of duplicate records, titles and abstracts were screened for relevance. Records that did not meet the predefined inclusion criteria were excluded.

**Figure 1:**
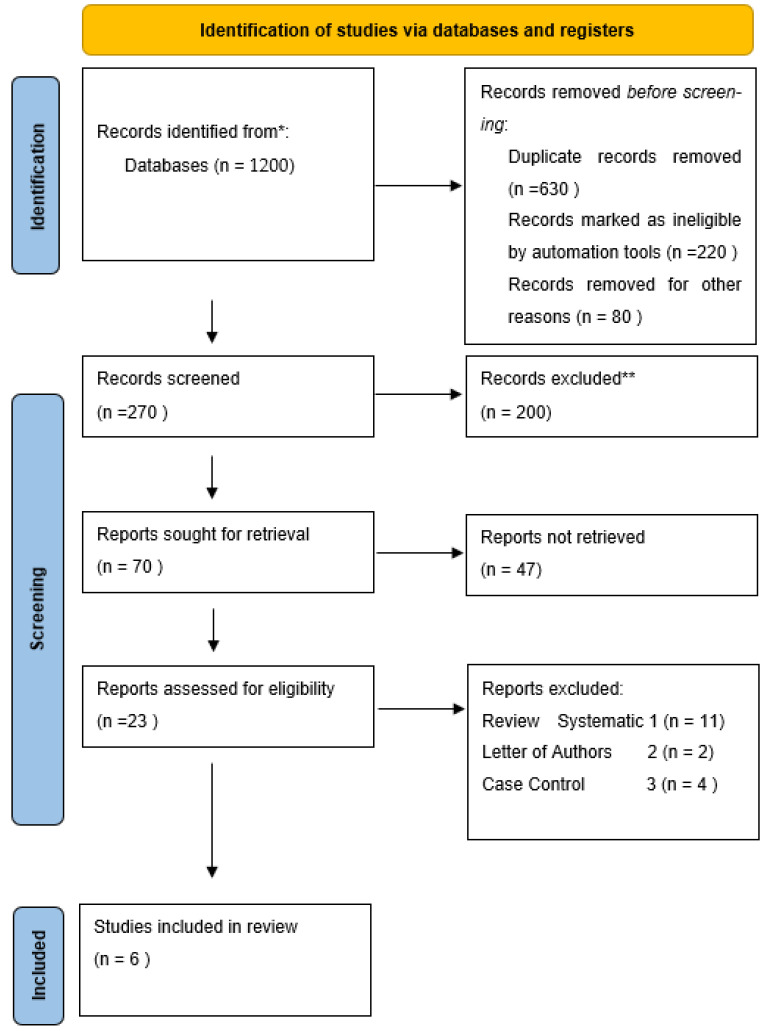
Prisma Flow Diagram.

Full-text articles were subsequently assessed for eligibility, and studies were excluded based on predefined criteria, including ineligible population, insufficient outcome data, or inappropriate study design. Ultimately, six observational studies met the eligibility criteria and were included in both the qualitative synthesis and quantitative synthesis (meta-analysis). The study selection process is summarized in the PRISMA flow diagram (Figure 1). Table S1: PRISMA 2020 Checklist see the Supplementary Materials.

### 2.3. Eligibility Criteria

Given the variability in diagnostic criteria across observational studies, both polysomnographic and validated questionnaire-based definitions of OSA were accepted, and this heterogeneity was accounted for in the random-effects model. Observational studies evaluating the association between obstructive sleep apnea (OSA) and gastroesophageal reflux disease (GERD) were considered eligible for inclusion. Studies were included if they met the following criteria: (1) adult human populations (≥18 years); (2) assessment of OSA diagnosed by polysomnography, validated questionnaires, or clinical criteria; (3) assessment of GERD, including symptomatic GERD, non-erosive reflux disease (NERD), or endoscopically confirmed disease; and (4) reporting of effect estimates as odds ratios (ORs) with corresponding 95% confidence intervals (CIs), or sufficient data to calculate them. Case reports, narrative reviews, editorials, conference abstracts without full data, pediatric studies, and animal studies were excluded [18].

### 2.4. Information Sources and Search Strategy

A comprehensive literature search was performed in major electronic databases, including PubMed/MEDLINE, Scopus, Web of Science, and Embase, from inception to the most recent search date. The search strategy combined controlled vocabulary and free-text terms related to obstructive sleep apnea and gastroesophageal reflux disease. Reference lists of included studies and relevant reviews were also manually screened to identify additional eligible articles.

### 2.5. Study Selection

All retrieved records were imported into a reference management software, and duplicate records were removed. Two reviewers independently screened titles and abstracts for eligibility. Full-text articles were subsequently assessed for inclusion based on the predefined criteria. Discrepancies were resolved through discussion and consensus. The study selection process is illustrated using a PRISMA flow diagram.

### 2.6. Data Extraction

Data were independently extracted by two reviewers using a standardized data extraction form. Extracted variables included study characteristics (author, year, country, study design), population characteristics, diagnostic criteria for OSA and GERD, sample size, effect estimates (ORs and 95% CIs), and variables included in adjusted analyses. When multiple models were reported, the most fully adjusted estimates were extracted.

### 2.7. Risk of Bias Assessment

The methodological quality and risk of bias of the included observational studies were assessed using the Newcastle–Ottawa Scale (NOS). This tool evaluates studies across three domains: selection of study groups, comparability of groups, and assessment of exposure or outcome. Studies scoring 7–9 points were considered high quality, 4–6 [19] points moderate quality, and ≤3 points low quality. Risk of bias assessment was conducted independently by two reviewers, with disagreements resolved by consensus. Given the small number of included studies, the ability to detect publication bias is limited.

### 2.8. Statistical Analysis

A meta-analysis was performed to estimate the pooled association between obstructive sleep apnea and gastroesophageal reflux disease. Odds ratios with 95% confidence intervals were synthesized using a random-effects model to account for between-study heterogeneity. Statistical heterogeneity was assessed using the I^2^ statistic, with values of 25%, 50%, and 75% representing low, moderate, and high heterogeneity, respectively. All statistical analyses were conducted using standard meta-analytical software.

## 3. Results

### 3.1. Quantitative Synthesis (Forest Plot)

A total of six observational studies were included in the final qualitative and quantitative synthesis. All studies employed a cross-sectional or case–control design and were conducted in diverse geographic settings, including the United States, South Korea, Turkey, and Poland. The study populations consisted primarily of adult patients evaluated for obstructive sleep apnea (OSA), gastroesophageal reflux disease (GERD), or both. Across the included studies, exposure variables included the presence or severity of OSA, GERD (including non-erosive reflux disease), or related metabolic factors, while outcomes focused on the association between OSA and GERD or related clinical manifestations (Table 1).

Most included studies reported adjusted effect estimates after controlling for key confounding variables. Commonly adjusted covariates included age, sex, body mass index, and obesity-related parameters, while some studies additionally accounted for lifestyle factors or metabolic markers. Adjusted odds ratios were primarily derived using multivariable logistic regression models. The variables included in the adjusted analyses and the statistical models employed in each study (Table 2).

The methodological quality of the included studies was assessed using the Newcastle–Ottawa Scale for observational studies. Overall, the risk of bias was judged to be low to moderate across studies. Most studies achieved high scores in the selection and outcome/exposure domains, reflecting appropriate participant selection and reliable outcome assessment. All studies demonstrated adequate comparability through adjustment for relevant confounders (Table 3).

**Figure 2:**
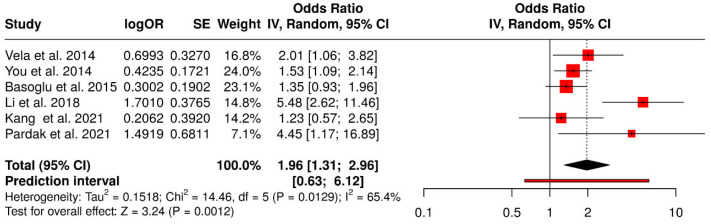
Metanalysis [3,5,20,22,23,24].

The forest plot integrating the six included observational studies demonstrated a positive association between obstructive sleep apnea and gastroesophageal reflux disease. Most individual studies reported odds ratios greater than 1, indicating increased odds of GERD or reflux-related outcomes among individuals with OSA. Using a random-effects model, the pooled analysis showed a statistically significant association, with a combined odds ratio (OR) of 1.96. The 95% confidence interval did not cross the null value, confirming the statistical significance of the association. These findings indicate that individuals with obstructive sleep apnea have approximately two-fold higher odds of presenting gastroesophageal reflux disease compared with those without OSA (Figure 2).

### 3.2. Heterogeneity Assessment

Moderate heterogeneity was observed among the included studies, with an I^2^ value of 65%, suggesting that a substantial proportion of the variability in effect estimates was attributable to between-study differences rather than chance alone. This level of heterogeneity supports the use of a random-effects model and may reflect variations in study populations, diagnostic criteria, disease severity, and adjustment for confounding factors such as obesity and age.

### 3.3. Publication Bias (Funnel Plot)

**Figure 3:**
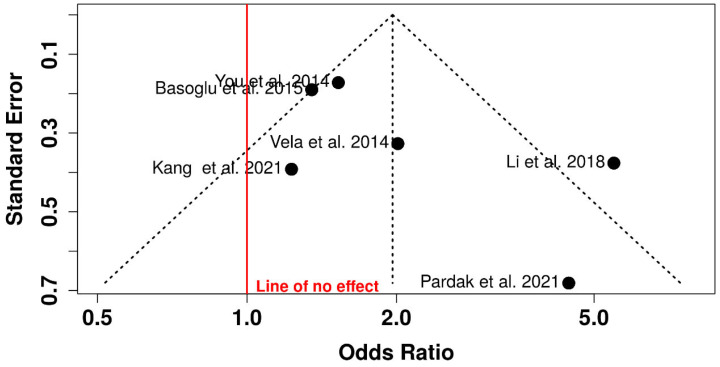
Funnel Plot of Metanalysis [3,5,20,22,23,24].

Visual inspection of the funnel plot showed an approximately symmetrical distribution of studies around the pooled effect estimate. Studies with lower precision demonstrated wider dispersion, while studies with higher precision clustered closer to the overall effect size. No substantial asymmetry suggestive of publication bias was observed. These findings indicate that the pooled association between obstructive sleep apnea and gastroesophageal reflux disease is unlikely to be significantly influenced by selective publication of studies with positive results (Figure 3).

## 4. Discussion

This systematic review and meta-analysis synthesized available observational evidence to evaluate the association between obstructive sleep apnea (OSA) and gastroesophageal reflux disease (GERD) in adult populations [20,22]. The pooled analysis demonstrated a statistically significant association, with individuals diagnosed with OSA presenting nearly two-fold higher odds of GERD compared to those without OSA (OR = 1.96). Although moderate heterogeneity was observed (I^2^ = 65%), the direction of effect was consistent across studies, suggesting a reproducible association across diverse populations and study designs [25].

The magnitude of the pooled estimate should be interpreted cautiously. Moderate heterogeneity indicates that between-study differences contributed substantially to variability in effect sizes [26]. These differences likely reflect variations in study populations, geographic settings, OSA diagnostic methods, GERD definitions, and adjustment strategies [21]. Nevertheless, the consistency in the direction of association across all included studies strengthens confidence that the observed relationship is unlikely to be attributable to random variation alone [27].

Several pathophysiological mechanisms may plausibly explain the association between OSA and GERD [28]. Recurrent obstructive events during sleep generate pronounced negative intrathoracic pressure, increasing the trans-diaphragmatic pressure gradient and facilitating retrograde movement of gastric contents into the esophagus. In addition, sleep fragmentation and intermittent hypoxia may contribute to autonomic dysregulation, potentially altering lower esophageal sphincter function and esophageal motility [29]. These mechanisms provide biological plausibility for the observed association; however, causality cannot be established based on the observational evidence synthesized in this review [30].

Obesity represents a central shared risk factor for both OSA and GERD and likely contributes to the observed relationship [31]. Increased body mass index and central adiposity promote upper airway collapsibility, elevate intra-abdominal pressure, and alter respiratory mechanics. Importantly, most included studies adjusted for obesity-related variables, and the association between OSA and GERD remained statistically significant in adjusted models [32]. However, residual confounding cannot be excluded, particularly given variability in covariate selection and measurement across studies [33]. Therefore, the present findings should not be interpreted as evidence of an independent causal relationship but rather as an adjusted association observed across multiple populations [34].

Heterogeneity in diagnostic criteria warrants careful consideration. OSA was diagnosed using polysomnography in some studies, while others relied on validated questionnaires or clinical assessments [35]. Similarly, GERD definitions varied and included symptom-based diagnoses, non-erosive reflux disease (NERD), and endoscopically confirmed disease. These differences may capture distinct phenotypes and severities of both conditions, potentially contributing to the observed I^2^ value [36]. The use of a random-effects model was therefore appropriate to account for this methodological diversity [37].

The funnel plot did not demonstrate marked asymmetry; however, the small number of included studies limits the statistical power to detect publication bias or small-study effects [38]. Consequently, the absence of apparent asymmetry should not be interpreted as definitive evidence of no publication bias. This limitation is inherent to meta-analyses with a limited number of eligible studies [39].

From a clinical perspective, the findings highlight the frequent coexistence of OSA and GERD in adult populations. While the current evidence does not justify formal screening recommendations, it supports increased clinical awareness of reflux symptoms in patients with OSA, particularly in those presenting with persistent nocturnal complaints or suboptimal response to therapy [40]. Similarly, clinicians managing GERD may consider evaluation for sleep-disordered breathing in selected high-risk patients [41].

This study has several strengths. It followed a predefined protocol registered in PROSPERO and adhered to PRISMA 2020 reporting standards [42]. A comprehensive search strategy was applied across multiple databases, and methodological quality was assessed using the Newcastle–Ottawa Scale [43]. The extraction of the most fully adjusted effect estimates enhances the validity of the pooled analysis [44].

Nevertheless, important limitations must be acknowledged. All included studies were observational in design, precluding causal inference and limiting assessment of temporal relationships [23]. The relatively small number of studies restricted the ability to perform subgroup analyses or meta-regression to explore sources of heterogeneity [45]. Diagnostic heterogeneity in both OSA and GERD definitions may affect generalizability. Additionally, geographic concentration of studies in specific regions may limit extrapolation to other populations with differing demographic or lifestyle characteristics [46,47].

Future research should prioritize prospective cohort studies and interventional trials to clarify temporal directionality and potential causal mechanisms [48]. Studies examining whether effective treatment of OSA, particularly with continuous positive airway pressure (CPAP), results in improvement of GERD outcomes may provide valuable insight into mechanistic pathways [24]. Standardized diagnostic criteria and harmonized outcome definitions will be essential to improve comparability and precision in future meta-analyses [48].

In summary, this systematic review and meta-analysis demonstrate a statistically significant association between obstructive sleep apnea and gastroesophageal reflux disease in adults. While moderate heterogeneity and methodological variability warrant cautious interpretation, the consistent direction of effect across studies suggests a meaningful clinical relationship that merits further investigation.

## 5. Conclusions

This meta-analysis indicates that obstructive sleep apnea is associated with nearly two-fold increased odds of gastroesophageal reflux disease in adults. These findings may justify increased clinical awareness rather than formal screening recommendations.

## 6. Limitations

Several limitations of the present systematic review and meta-analysis should be acknowledged. First, all included studies were observational in design, which inherently limits the ability to establish causal relationships between obstructive sleep apnea and gastroesophageal reflux disease. Although adjusted analyses were used in most studies, residual confounding cannot be fully excluded. Second, moderate heterogeneity was observed across studies (I^2^ = 65%), indicating variability in effect estimates that may be attributable to differences in study populations, diagnostic criteria, and methodological approaches. In particular, heterogeneity in the assessment of obstructive sleep apnea—ranging from polysomnography to questionnaire-based or clinical diagnoses—and variability in GERD definitions (symptom-based versus endoscopic diagnosis) may have influenced the pooled results. Third, the number of included studies was relatively small, which may limit the precision of the pooled effect estimate and reduce the statistical power to explore sources of heterogeneity through subgroup or meta-regression analyses. Additionally, the limited number of studies restricts the ability to definitively rule out small-study effects or subtle publication bias, despite the absence of marked asymmetry in the funnel plot. Fourth, most studies were conducted in specific geographic regions, which may affect the generalizability of the findings to other populations with different demographic, lifestyle, or healthcare characteristics. Differences in obesity prevalence, dietary habits, and access to diagnostic testing may further influence the observed association between OSA and GERD. Finally, the cross-sectional nature of several included studies precludes assessment of temporal relationships. As a result, it remains unclear whether obstructive sleep apnea predisposes individuals to gastroesophageal reflux disease, whether GERD contributes to sleep-disordered breathing, or whether both conditions arise from shared underlying risk factors. Despite these limitations, the present meta-analysis provides a comprehensive synthesis of available evidence and offers clinically relevant insights into the association between obstructive sleep apnea and gastroesophageal reflux disease.

## Figures and Tables

**Table 1:** General characteristics of the included studies.

Author	Year	Country	Design	Population	N Total	Exposure	Primary Outcome
Basoglu et al. [5]	2015	Türkiye	Case–control studies	Adults with OSA	186	GERD	Presence/OSA severity
Kang et al. [20]	2021	South Korea	Cross-sectional observational	OSA Patients	NR	GERD	Daytime sleepiness
Kim et al. [21]	2018	South Korea	Cross-sectional observational	OSA Patients	NR	OSA Severity	Endoscopic GERD
Pardak et al. [22]	2021	Poland	Cross-sectional observational	Adults with OSA	NR	Metabolic hormones	
Vela et al. [23]	2014	USA	Cross-sectional observational	Adults with Sleep Symptoms	NR	OSA	Sleep Quality/OSA
You et al. [24]	2014	South Korea	Cross-sectional population study	General Population	NR	NERD	High risk of OSA

**Table 2:** Settings and Confounding Variables.

Author’s	Adjusted Variables	Adjusted OR	Statistical Model
Basoglu et al. [5]	Obesity, sex	Adjusted OR	Logistic regression
Kang et al. [20]	Age, sex, BMI	Adjusted OR	Logistic regression
Kim et al. [21]	BMI, severity OSA	Adjusted OR	Logistic regression
Pardak et al. [22]	BMI, metabolic markers	Adjusted OR	Logistic regression
Vela et al. [23]	Age, sex, BMI	Adjusted OR	Logistic regression
You et al. [24]	Age, sex, BMI, smoking	Adjusted OR	Logistic regression

**Table 3:** Risk of Bias—Newcastle-Ottawa Scale (NOS).

Authors	Selection (4)	Comparability (2)	Outcome (3)	Total	Quality
Basoglu et al. [5]	★★★★	★★	★★★	9	High
Kang et al. [20]	★★★	★★	★★	7	High
Kim et al. [21]	★★★	★★	★★	7	High
Pardak et al. [22]	★★★★	★★	★★★	9	High
Vela et al. [23]	★★★	★★	★★	7	High
You et al. [24]	★★★★	★★	★★	8	High

## Data Availability

Not applicable.

## Supplementary Materials

The following supporting information can be downloaded at: https://www.mdpi.com/article/10.3390/jcm15072518/s1, Table S1: PRISMA 2020 Checklist [29].

## Abbreviation

Abbreviation	Meaning
OSA	Obstructive Sleep Apnea
GERD	Gastroesophageal Reflux Disease
NERD	Non-Erosive Reflux Disease
OR	Odds Ratio
CI	Confidence Interval
BMI	Body Mass Index
NOS	Newcastle–Ottawa Scale
RS	Systematic Review
MA	Meta-analysis
PRISMA	Preferred Reporting Items for Systematic Reviews and Meta-Analyses
AHI	Apnea–Hypopnea Index
IMC	Body Mass Index
I^2^	Higgins heterogeneity statistic
NR	Not Reported

## References

[1] Benjafield A., Ayas N.T., Eastwood P.R. (2019). Estimation of the global prevalence of obstructive sleep apnoea. Lancet Respir. Med..

[2] Senaratna C.V., Perret J., Lodge C.J. (2017). Prevalence of obstructive sleep apnea in the general population: A meta-analysis. Sleep Med. Rev..

[3] Li R., Xia J., Zhang X.I., Gathirua-Mwangi W.G., Guo J., Li Y., McKenzie S., Song Y. (2018). Associations of Muscle Mass and Strength with All-Cause Mortality among US Older Adults. Med. Sci. Sports Exerc..

[4] Peppard P.E., Young T., Barnet J.H. (2013). Increased prevalence of sleep-disordered breathing in adults. Am. J. Respir. Crit. Care Med..

[5] Basoglu O.K., Sarac S., Vardar R., Tasbakan M.S., Ucar Z.Z., Ayik S., Kose T., Bor S. (2015). Obstructive sleep apnea syndrome and gastroesophageal reflux disease: The effect of apnea severity. Sleep Breath..

[6] El-Serag H.B., Sweet S., Winchester C., Dent J. (2014). Update on the epidemiology of GERD. Gut.

[7] Eusebi L., Ratnakumaran R., Yuan Y. (2018). Global prevalence of GERD: A systematic review and meta-analysis. Gut.

[8] Vakil N., van Zanten S., Kahrilas P. (2006). The Montreal definition and classification of GERD. Am. J. Gastroenterol..

[9] Maret-Ouda J., Markar S.R., Lagergren J. (2020). Gastroesophageal reflux disease: A review. Lancet.

[10] Emilsson Ö.I., Aspelund T., Janson C., Benediktsdottir B., Juliusson S., Maislin G. (2024). Nocturnal gastro-oesophageal reflux and respiratory symptoms are increased in sleep apnoea: Comparison with the general population. BMJ Open Respir. Res..

[11] Green B., Broughton W., O’Connor J.B. (2003). Intrathoracic pressure swings promote reflux in OSA. Am. J. Gastroenterol..

[12] Fabozzi A., Bouloukaki I., Bonini M., Schiza S., Palange P. (2025). Asthma-OSA overlap syndrome: A distinct endophenotype?. Respir. Med..

[13] Sgaria V.P., Cielo C.A., Bortagarai F.M., Fleig A.H.D., Callegaro C.C. (2024). CPAP Treatment Improves Quality of Life and Self-perception of Voice Impairment in Patients with OSA. J. Voice.

[14] Peker Y., Akdeniz B., Altay S., Balcan B., Başaran Ö., Baysal E. (2023). Obstructive Sleep Apnea and Cardiovascular Disease: Where Do We Stand?. Anatol. J. Cardiol..

[15] Messineo L., Bakker J.P., Cronin J., Yee J., White D.P. (2024). Obstructive sleep apnea and obesity: A review of epidemiology, pathophysiology and the effect of weight-loss treatments. Sleep Med. Rev..

[16] Orr W.C., Fass R., Sundaram S., Scheimann A.O. (2020). The effect of sleep on gastrointestinal functioning in common digestive diseases. Lancet Gastroenterol. Hepatol..

[17] Bordoni B., Morabito B. (2026). Rating Scales for Obstructive Sleep Apnea Syndrome: The Importance of a Comprehensive Assessment. Cureus.

[18] Boira I., Chiner E. (2025). Sleep and Respiratory Infections. Semin. Respir. Crit. Care Med..

[19] Fass R., Boeckxstaens G., El-Serag H., Rosen R., Sifrim D., Vaezi M. (2021). Gastro-oesophageal reflux disease. Nat. Rev. Dis. Primers.

[20] Kang S., Kim J., Lee S. (2021). GERD and excessive daytime sleepiness in OSA. J. Clin. Sleep Med..

[21] Kim Y., Lee Y.J., Park J.S., Cho Y.J., Yoon H.I., Lee J.H., Lee C.T., Kim S.J. (2018). Relationship between obstructive sleep apnea severity and endoscopic gastroesophageal reflux disease. Sleep Med..

[22] Pardak P., Wysocki J., Białasiewicz P. (2021). Metabolic alterations in OSA. Endokrynol. Pol..

[23] Vela M.F. (2014). Gastroesophageal reflux disease and sleep disturbances: A population-based study. Clin. Gastroenterol. Hepatol..

[24] You C.R., Oh J.H., Kim J.Y. (2014). Gastroesophageal reflux disease is associated with obstructive sleep apnea in the general population. J. Neurogastroenterol. Motil..

[25] Mechanick J., Apovian C., Brethauer S., Garvey W., Joffe A., Kim J. (2019). Clinical Practice Guidelines for the Perioperative Nutrition, Metabolic and Non surgical Support of Patients undergoin bariatric procedures 2019 Update: Cosponsored by American Association of Clinical Endocrinologists/American College of Endocrinology, The Obesity Society. Endocr. Pract..

[26] Pappa A., Muschaweck M., Wenzl T.G. (2023). Change of Sleep Stage during Gastroesophageal Reflux in Infants. Children.

[27] Torres G., Sánchez de la Torre M., Pinilla L., Barbé F. (2024). Obstructive sleep apnea and cardiovascular risk. Clin. Investig. Arter..

[28] Dicker D., Karpati T., Promislow S., Reges O. (2025). Implications of the European Association for the Study of Obesity’s New Framework Definition of Obesity: Prevalence and Association With All-Cause Mortality. Ann. Intern. Med..

[29] Page M.J., McKenzie J.E., Bossuyt P. (2021). PRISMA 2020 statement. BMJ.

[30] Castillejos-López M., Romero Y., Varela-Ordoñez A., Flores-Soto E., Romero-Martinez B.S., Velázquez-Cruz R., Vázquez-Perez J.A., Ruiz V., Gomez-Verjan J.C., Rivero-Segura N.A. (2023). Hypoxia Induces Alterations in the Circadian Rhythm in Patients with Chronic Respiratory Diseases. Cells.

[31] Higgins J., Thompson S., Deeks J., Altman D. (2003). Measuring inconsistency in meta-analyses. BMJ.

[32] Fanelli D., Costas R., Ioannidis J. (2017). Meta-assessment of bias in science. Proc. Natl. Acad. Sci. USA.

[33] Carra M., Romandini P., Romandini M. (2025). Risk of Bias Evaluation of Cross-Sectional Studies: Adaptation of the Newcastle-Ottawa Scale. J. Periodontal. Res..

[34] Migliavaca C.B., Stein C., Colpani V., Barker T.H., Ziegelmann P.K., Munn Z., Falavigna M. (2022). Prevalence Estimates Reviews-Systematic Review Methodology Group (PERSyst). Meta-analysis of prevalence: I^2^ statistic and how to deal with heterogeneity. Res. Synth. Methods.

[35] Spranger J., Homberg A., Sonnberger M., Niederberger M. (2022). Reporting guidelines for Delphi techniques in health sciences: A methodological review. Z Evid. Fortbild. Qual. Gesundhwes.

[36] Cumpston M.S., McKenzie J.E., Welch V.A., Brennan S.E. (2022). Strengthening systematic reviews in public health: Guidance in the Cochrane Handbook for Systematic Reviews of Interventions, 2nd edition. J. Public Health.

[37] Sands S.A., Edwards B., Terrill P.I. (2014). Mechanisms of OSA. J. Appl. Physiol..

[38] van Zeller M., McNicholas W. (2024). Sleep disordered breathing: OSA-COPD overlap. Expert Rev. Respir. Med..

[39] Dent J., El-Serag H.B., Wallander M.A., Johansson S. (2005). Epidemiology of GERD. Gut.

[40] Sunwoo B., Raphelson J., Malhotra A. (2024). Chronic obstructive pulmonary disease and obstructive sleep apnea overlap: Who to treat and how?. Expert Rev. Respir. Med..

[41] Zhang D., Liu S., Li Z., Wang R. (2022). Global, regional and national burden of gastroesophageal reflux disease, 1990-2019: Update from the GBD 2019 study. Ann. Med..

[42] Wang Y., Liu K., Hu K. (2020). OSA and reflux severity meta-analysis. Sleep Med..

[43] Patel D.A., Naik R.D., Slaughter J.C. (2015). GERD symptom burden. Clin. Gastroenterol. Hepatol..

[44] Gileles-Hillel A., Bhattacharjee R., Gorelik M., Narang I. (2024). Advances in Sleep-Disordered Breathing in Children. Clin. Chest Med..

[45] Lyons M., Bhatt N., Pack A., Magalang U. (2020). Global burden of sleep-disordered breathing and its implications. Respirology.

[46] Becker N., Kalpouzos G., Salami A., Laukka E., Brehmer Y. (2019). Structure-function associations of successful associative encoding. Neuroimage.

[47] Davarpanah Jazi A., Shahabi S., Sheikhbahaei E., Tolone S., Skalli M., Kabir A. (2023). A systematic review and meta-analysis on GERD after OAGB: Rate, treatments, and success. Expert Rev. Gastroenterol. Hepatol..

[48] Ioannidis J. (2022). Correction: Why Most Published Research Findings Are False. PLoS Med..

